# MR-guided focused ultrasound (MRgFUS) is effective for the distinct pattern of uterine fibroids seen in African-American women: data from phase III/IV, non-randomized, multicenter clinical trials

**DOI:** 10.1186/2050-5736-1-23

**Published:** 2013-12-02

**Authors:** Ronit Machtinger, Fiona M Fennessy, Elizabeth A Stewart, Stacey A Missmer, Katharine F Correia, Clare MC Tempany

**Affiliations:** 1 Department of Obstetrics, Gynecology and Reproductive Biology, Brigham and Women’s Hospital and Harvard Medical School, 75 Francis St, Boston, MA 02115, USA; 2 Department of Obstetrics and Gynecology, Sheba Medical Center, Ramat Gan 52561, Israel; 3 Department of Radiology, Brigham and Women’s Hospital and Harvard Medical School, 45 Francis St., Boston, MA 02115, USA; 4 Department of Obstetrics and Gynecology, Mayo Medical School, Mayo Clinic, Rochester, MN 55905, USA; 5 Channing Division of Network Medicine, Department of Medicine, Brigham and Women’s Hospital and Harvard Medical School, Boston, MA 02115, USA; 6 Department of Epidemiology, Harvard School of Public Health, Boston, MA 02115, USA

**Keywords:** MRgFUS, Fibroids, Leiomyoma, African-American, Alternative treatment

## Abstract

**Background:**

Uterine fibroids are common among women at the reproductive age. Magnetic resonance-guided focused ultrasound surgery (MRgFUS) is a novel and a conservative treatment for symptomatic cases. The aim of the study was to evaluate the efficacy of MRgFUS in African-American (AA) women compared with that in non-African-Americans (non-AA).

**Methods:**

A single-armed phase IV study was conducted to establish the efficacy of treatment in AA women. Comparison of patient, fibroid, and treatment characteristics from this trial was compared with that of the previously published phase III trial. Both studies were approved by the IRB of each medical center.

**Results:**

Sixty-three AA and 59 non-AA women were treated with MRgFUS. Although AA women had a different pattern of disease, outcomes were similar in both groups. AA patients had a significant higher total number of fibroids compared with non-AA (median 6.0, interquartile range (IQR) 3.0–10.0 vs. 2.0, IQR 1.0–4.0, respectively, *p* < 0.001), although their total fibroid volume was significantly smaller (median 196.9 cm^3^, IQR 112.8–415.3 cm^3^ vs. 394.8 cm^3^, IQR 189.8–674.4 cm^3^, respectively, *p* < 0.001). AA women were younger compared with non-AA (mean ± SD 43.4 ± 5.1 vs. 46.3 ± 4.1 years of age, respectively, *p* = 0.001) when they presented for treatment. The rate of alternative treatments as well as fibroid-associated symptoms at follow-up time points (3, 6, 12, 24, and 36 months, period following MRgFUS treatment) did not differ according to race (*p* ≥ 0.62).

**Conclusion:**

Despite differences in the pattern of fibroid disease, MRgFUS for uterine fibroids has a similar efficacy for AA women compared with non-AA women.

## Background

Uterine fibroids (leiomyomas or myomas) are an important healthcare issue, chiefly because of their frequency, associated morbidity, and their contribution to the rate of hysterectomy [1,2]. Hysterectomy rates have declined in recent years in part due to the increasing array of minimally invasive alternatives to hysterectomy utilized for fibroid treatment including laparoscopic, hysteroscopic, and robotic myomectomy, endometrial ablation, uterine artery embolization (UAE), and magnetic resonance imaging (MRI)-guided focused ultrasound surgery (MRgFUS) as well as medical treatments [3,4].

Uterine fibroids have significant health disparities for African-American (AA) women. Compared to other women, AA women have evidence of a more extensive pattern of fibroids and an earlier age of onset [5-8]. Genetic and biologic differences and differential growth rates may contribute to this racial disparity [9-12]. Despite this disparity, most studies of fibroid treatment have not reported the racial composition of enrolled participants, and studies that do report race often have few AA women [13].

MRgFUS is a Food and Drug Administration (FDA)-approved therapy for uterine leiomyomas that has been demonstrated in multiple reports to result in sustained treatment efficacy [14-20]. Early cohorts studied with this treatment were largely non-AA. In light of this reality, the primary purposes of this report were to evaluate the efficacy of MRgFUS in AA women relative to non-AA women and to determine the risk of adverse effects in the AA population. A secondary goal was to compare the pattern of fibroids in AA women enrolled in this study to that in other women enrolled in a previous clinical trial of MRgFUS that had the same enrollment criteria [21].

## Methods

All data on AA women was collected as part of a phase IV, non-randomized, multicenter clinical trial designed to evaluate the safety and effectiveness of MRgFUS treatment in this specific high-risk population. Funding was provided by the device manufacturer (InSightec, Haifa, Israel), and the protocol was approved by each local institutional review board.

Women were treated between January 2005 and April 2006 at one of nine US clinical centers (Brigham and Women’s Hospital (BWH), Boston, MA, USA; Mayo Clinic, Rochester, MN, USA; Johns Hopkins Hospital, Baltimore, MD, USA; Radnet, Beverly Hills, CA, USA; Lahey, Burlington, MA, USA; Sightline, Houston, TX, USA; North Texas Uterine Fibroid Institute, Dallas, TX, USA; UMRI Boca Raton, FL, USA; and Virtua, Voorhees, NJ, USA). Because the enrollment criteria and treatment guidelines were identical to the earlier phase III multicenter clinical trial that led to the approval of this device, we utilized, as a comparison group, the data from this published clinical trial, whose patients were treated between April 2004 and January 2005 [21]. Both clinical studies were approved by the IRB from each site.

The eligibility criteria for both trials have been previously described [14,19,21]. Briefly, subjects were premenopausal women in good general health, with symptomatic uterine fibroids, and who had no future childbearing plans.

### Enrollment and screening

Patients seeking treatment for symptomatic uterine fibroids at one of nine US clinical centers were recruited through the gynecology and interventional radiology clinics. Flyers, posters, and local advertisements were used as recruitment tools. Eligible premenopausal women were asked to complete a validated Symptom Severity Score (SSS) from the uterine fibroid symptom and quality of life (UFS-QOL) questionnaire [22]. The scores were transformed to create a transformed SSS, yielding a 0 to 100 point scale with higher scores indicating worse symptoms [22]. Patients with SSS ≥ 41 (i.e., symptomatic uterine fibroids) were scheduled for a medical and gynecological assessment. If there were no significant medical contraindications for treatment (such as claustrophobia, severe hypertension, severe cerebrovascular disease, severe anemia, clotting disorders, or MR contraindications such as a cardiac pacemaker or other metallic devices, significant anterior abdominal scarring, high BMI, etc.), a pelvic MRI with intravenous gadolinium contrast was performed. MR images were analyzed to determine the number, size, and location of the fibroids. The enhancement patterns were also assessed on the post-contrast images. Screen failures were defined as women with symptomatic uterine fibroids who were interested in MRgFUS treatment but were not eligible due to one of the following: inadequate fibroid-associated symptoms, a medical history that was a contraindication for the treatment or imaging-associated reasons, or MR findings such as necrotic fibroid, extensive adenomyosis, multiple small (<3 cm) fibroids or single fibroids over 8 cm, and ones with large bowel loops interposed between the uterus and anterior abdominal wall.

### Equipment

The MRgFUS system (ExAblate 2000, InSightec, Haifa, Israel) integrates a standard 1.5-T MRI system (GE Medical Systems, Milwaukee, WI, USA) with the focused ultrasound treatment transducer housed in a water bath within the MR table. The MR imaging system, described in detail by Tempany et al. [20] and Stewart et al. [14], provides real-time feedback of the temperature changes by incorporating MR thermometry that uses sensitive imaging sequences during each sonication.

### Patient preparation and treatment

Patient preparation has been previously described [20,21,23]. T2-weighted pelvic MR images were acquired and used for treatment planning. A region of treatment was defined within the fibroid on coronal imaging, and the number of spots (sonications) required to ablate this volume was calculated by the treatment software. Each sonication of focused ultrasound energy targeted an approximately 1-cm^3^ bean-shaped region. The sonication could be altered in power, frequency, diameter, and length to allow a great treatment volume as safely as possible using as few sonications as possible. Once planning was complete, low-energy test sonications were delivered to confirm correct sonication location. The power was then increased until the temperature-sensitive maps demonstrated a therapeutic thermal focus. Treatment failure was defined as a treatment in which fewer than ten sonications were performed.

At the end of the treatment, all patients underwent a contrast-enhanced T1-weighted MRI with IV administration of gadolinium (gadopentetate dimeglumine) and the resultant volume of non-enhancing tissue (non-perfused volume (NPV)) was measured. The NPV ratio to treated fibroid volume and NPV ratio to total fibroid volume were determined. In cases of patients who underwent two treatments, the NPV in the study was the total NPV achieved following the second MRgFUS treatment. The volume of treated fibroids was calculated from MRI images at 6 and 12 months after the treatment and compared with baseline.

### Treatment protocols

The goal of treatment was to deliver the therapeutic sonications to as large fibroid area as possible, within the limitation of the clinical trial rules. All patients were treated according to similar guidelines, as outlined by Fennessy et al. [21]. Treatment was limited to 50% of fibroid volume (except 33% if submucosal) or 150-cm^3^ maximum volumes. Maximum treatment time (from the first to the last sonication) was 180 min. In cases where a second treatment was deemed necessary, the second treatment was performed within a 14-day period.

### Data collection

Patient’s age, body mass index (BMI; kg/m^2^), race, total fibroid number, total fibroid volume (cm^3^), and volume of the treated fibroid(s) (cm^3^) were all prospectively collected. The patient’s race was self-defined, in keeping with the FDA’s recommendation, for the collection of race and ethnicity data in clinical trials [24]. Patients were categorized as AA if it was their only declared race. All others were classified as non-AA. Patients were followed for up to 36 months after treatment. At 1 week and 3 months post-procedure, a phone interview was conducted, and at 6, 12, 24, and 36 months, a face-to-face interview was conducted to obtain an updated UFS-QOL SSS score. Also, at 6 and 12 months, a clinical evaluation and pelvic MRI were obtained to measure the volume of the treated fibroids.

### Adverse events

All adverse events (AEs) were reported to the institutional review board and the US Food and Drug Administration as required. AEs were reported by the patients and then classified as serious or non-serious. Serious AEs were defined according to the Standard Code of Federal Regulation definitions for use of an investigational device [25]. In cases of non-serious AEs, the reported complaints were further classified as important (lasting for >14 days) or not important (lasting for less than 14 days).

### Screen failures

A subanalysis of the patients who were declared screen failures was conducted to compare the incidence and reasons for screen failure between AA and non-AA women using the data from the BWH site only (*N* = 39).

### Statistical analysis

Statistical analyses were conducted using Statistical Analysis Software (SAS®) version 9.1 (SAS Institute, Inc., Cary, NC, USA). The Mann–Whitney-Wilcoxon test, a two-sided non-parametric test for two independent samples, was used to study unadjusted differences in baseline and treatment characteristics between the two racial groups. To compare each group’s trajectory of outcomes over time, the correlation between measures from the same individual must be accounted for. Therefore, a generalized estimating equation with a binomial distribution, a logit link, and compound symmetry covariance structure were used for dichotomous outcomes (alternative treatments), and mixed models with an unstructured covariance structure were used for continuous outcomes (QOL score, volume of treated fibroids). The models were adjusted for age and BMI.

A chi-square test was used to compare the proportion of AEs in each group. Among women with at least one AE, the numbers of AEs were compared using the Mann–Whitney-Wilcoxon test. Data are presented as mean ± SD for normally distributed variables and median and 25th–75th percentile (interquartile range (IQR)) for non-normally distributed variables.

## Results and discussion

### Patients

One hundred and thirty-seven women (mean age 44.8 ± 4.8, range 34–53 years) comprised our study population, all of whom had MRgFUS fibroid treatment. There were 64 patients included from the phase III comparison study, 62 of these were non-AA. The remaining 73 women were from the phase IV AA study. Fifteen patients were excluded from the final analysis: four were treatment failures, two AA patients who were treated as part of the phase III study, four patients had adenomyosis, and five patients had missing data (one patient had missing treatment MRI data, two patients had missing post-treatment data, and two patients had all imaging data missing).

The final eligible study population comprised 122 women. Of these, 63 were AA and 59 non-AA. Among the non-AA women, 58 defined themselves as white and one as Hispanic. The AA women were significantly younger than the non-AA (mean ± SD 43.4 ± 5.1 vs. 46.3 ± 4.1 years of age, *p* = 0.001) and had a significantly higher BMI (27.7 ± 4.9 kg/m^2^ vs. 25.9 ± 4.2 kg/m^2^, *p* = 0.03). The AA women also had a significantly higher total number of fibroids (median 6.0, IQR 3.0–10.0 vs. 2.0, IQR 1.0–4.0, respectively). Total fibroid volume of the AA compared to the non-AA was smaller (median 196.9 cm^3^, IQR 112.8–415.3 cm^3^ vs. 394.8 cm^3^, IQR 189.8–674.4 cm^3^, *p* < 0.001) (Table 1). Baseline SSS QOL did not significantly differ between the groups.

**Table 1:** Demographic and treatment characteristics of AA and non-AA women with symptomatic uterine fibroids treated by MRgFUS

**Characteristics**	**AA (*n *** **= 63)**	**Non-AA (*n *** **= 59)**	***p *** **value**^**a**^
Age			0.001
Mean (SD)	43.4 (5.1)	46.3 (4.1)	
Median (Q1–Q3)	44.0 (40.0–47.0)	46.0 (44.0–49.0)	
Min.–max.	34.0–53.0	38.0–53.0	
Body mass index (kg/m^2^)			0.03
Mean (SD)	27.7 (4.9)	25.9 (4.2)	
Median (Q1–Q3)	27.3 (24.1–30.6)	25.3 (22.5–28.4)	
Min.–max.	18.2–40.7	19.1–39.5	
Total number of fibroids			<0.001
Mean (SD)	7.2 (5.4)	2.8 (2.3)	
Median (Q1–Q3)	6.0 (3.0–10.0)	2.0 (1.0–4.0)	
Min.–max.	1.0–30.0	1.0–11.0	
Total volume of fibroids (cm^3^)			<0.001
Mean (SD)	273.0 (216.0)	449.6 (299.6)	
Median (Q1–Q3)	196.9 (112.8–415.3)	394.8 (189.8–674.4)	
Min.–max.	15.7–807.2	18.1–1518.4	
Number of treated fibroids			0.01
Mean (SD)	1.6 (0.8)	1.3 (0.6)	
Median (Q1–Q3)	1.0 (1.0–2.0)	1.0 (1.0–1.0)	
Min.–max.	1.0–4.0	1.0–4.0	
Total volume of treated fibroids (cm^3^)			<0.001
Mean (SD)	177.9 (162.4)	408.5 (286.6)	
Median (Q1–Q3)	130.0 (64.6–235.0)	366.6 (183.7–564.0)	
Min.–max.	15.7–700.0	18.1–1518.4	
NPV ratio per total fibroid			0.52
Mean (SD)	28.2 (20.0)	26.3 (20.7)	
Median (Q1–Q3)	26.0 (15.3–41.0)	21.0 (9.0–42.0)	
Min.–max.ds	0.0–81.4	0.0–79.0	
NPV ratio per treated fibroid			0.03
Mean (SD)	40.6 (25.4)	29.9 (23.8)	
Median (Q1-Q3)	37.6 (25.6–55.1)	27.3 (9.3–48.0)	
Min.–max.	0.0–91.2	0.0–94.0	

^a^Two-sided Mann–Whitney-Wilcoxon *p* values. *NPV* non-perfused volume.

### Treatment volumes

**Figure 1:**
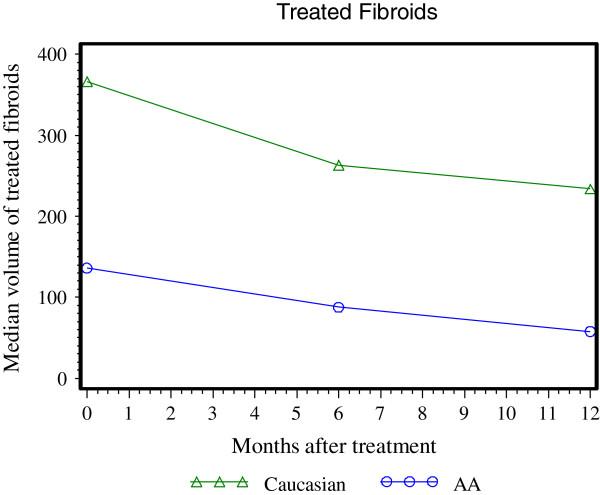
Median volume of treated fibroids at baseline and 6 and 12 months after MRgFUS treatment.

The volume of treated fibroids was significantly lower among the AA compared with the non-AA (177.9 ± 162.4 cm^3^ vs. 408.5 ± 286.6 cm^3^, *p* < 0.0001). The mean number of treated fibroids (per patient) was higher among the AA compared with non-AA patients (1.6 ± 0.8 vs. 1.3 ± 0.6, *p* = 0.01) (Table 1). Although the NPV ratio per treated fibroid was higher among AA women (40.6% ± 25.4% vs. 29.9% ± 23.8%, *p* = 0.03), there was no significant difference in the mean NPV per total fibroid volume at the end of treatment. The volume of treated fibroids at 6 and 12 months after treatment and compared with baseline is shown in Figure 1. Regardless of race, the mean volume of treated fibroids decreased significantly over time (*p* < 0.0001 for the AA and *p* = 0.002 for the non-AA).

### Adverse events

A total of 128 AEs were recorded for the 65 patients in both studies: 63 AEs were reported among AA and 65 AEs were reported among non-AA. No serious AEs were reported. Non-AA patients reported more AEs following treatment compared with AA. Among the non-AA, 47/59 (79.7%) reported at least one AE vs. 18/63 (28.6%) reported in AA patients (*p* < 0.0001). Among those who reported AEs, there was no significant difference in the number of AEs per patient reported (2.0 ± 1.5 and 1.8 ± 1.0, non-AA vs. AA).

The common types of AEs reported for both groups were back/leg pain (21.9%), abdominal cramping (21.1%), urinary tract infection/irritation symptoms (11.7%), gastrointestinal complaints (11.7%), skin irritation (7.0%), and vaginal bleeding or discharge (6.3%).

### Clinical outcome

**Figure 2:**
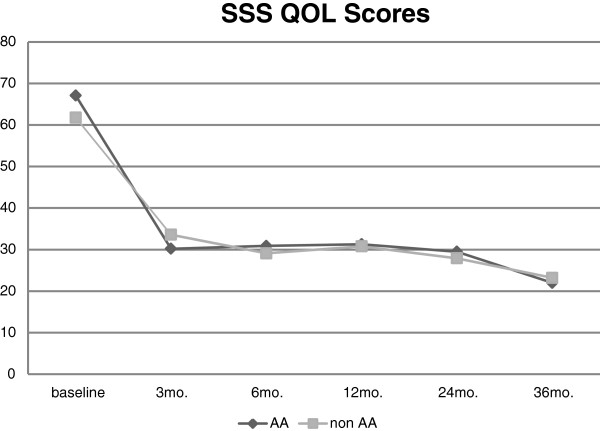
Change of transformed SSS QOL scores over time among AA and non-AA who were treated by MRgFUS.

One hundred and twenty patients (98%), 111 (91%), 91 (74.6%), 64 (52.4%), and 49 (40.2%) were followed to obtain their transformed SSS scores at 3, 6, 12, 24, and 36 months, respectively. The transformed SSS score significantly decreased over time for both groups of patients (both *p* < 0.0001). For the AA patients, median (IQR) transformed SSS scores were 68.8 (50.0–81.3) at baseline, 32.8 (12.5–46.9) at 12 months, 28.1 (9.4–43.8) at 24 months, and 12.5 (6.3–37.5) after 36 months. For the non-AA patients, transformed SSS scores were 59.4 (50.0–78.1) at baseline, 31.3 (18.8–43.8) at 12 months, 26.6 (9.4–43.8) at 24 months, and 18.8 (12.5–40.6) after 36 months. There was no difference between the groups for the rate of transformed QOL decrease (*p* = 0.62; Figure 2). Transformed SSS at 36 months was static, although data were available only for a small number of patients. The accumulated alternative treatments at the 6, 12, 24, and 36 months period following MRgFUS treatment were 4/115 (3.5%), 12/103 (11.7%), 32/96 (33.3%), and 42/91 (46.2%), respectively. The rate of alternative treatments at follow-up time points did not differ according to race (Table 2).

**Table 2:** Alternative treatments following MRgFUS treatment among AA and non-AA according to time of follow-up

**Months post-MRgFUS**	**Number of alternative treatments/total number of AA patients followed**	**Number of alternative treatments/total number of non-AA patients followed**	***p *** **value**^**a**^
3 months post-tx	1/62 (1.6%)	0/59 (0.0%)	0.98
6 months post-tx	1/59 (1.7%)	3/56 (5.4%)	
12 months post-tx	5/51 (9.8%)	7/52 (13.5%)	
24 months post-tx	18/48 (37.5%)	14/48 (29.2%)	
36 months post-tx	23/46 (50.0%)	19/45 (42.2%)	

^a^Two-sided *p* value from the generalized estimating equation (GEE) with a binomial distribution, a logit link, and a compound symmetry covariance structure (to account for the correlation between follow-ups from the same woman), testing whether the odds of seeking alternate treatments are different between races (separate but parallel lines hypothesis), given follow-up time, age, and BMI. *tx* treatment.

### Analysis of screen failures in a subpopulation

In a subanalysis, we compared the rates and the reasons for screen failures between AA women and non-AA women who sought MRgFUS treatment as a part of these clinical trials at a single institution. A total of 94 subjects provided informed consent for MRgFUS studies (40 AA and 54 non-AA) at the BWH site. Thirty-nine women (41.4%) failed to meet one or more enrollment criteria and were deemed screen failures.

AA women were more likely to be screen failures (52.5% AA vs. 33.3% non-AAs, *p* = 0.06). The reasons for screen failure differed significantly between groups. AA patients were more likely to fail the screening due to MRI findings that indicated that safe delivery of treatment was not possible, such as significant anterior abdominal wall scarring or bowel in the anterior ultrasound beam path (76.2% AA vs. 38.9% non-AA). However, they were less likely to fail screening due to inadequate fibroid-associated symptoms compared with the non-AA population (9.5% non-AA vs. 44.4% AA). Among the screen failures, AA patients had a higher mean number of fibroids at screening MRI (11.7 ± 11.8 AA vs. 4.4 ± 3.5 non-AA, *p* = 0.05) and their fibroid diameter was significantly smaller (median 2.4 cm, IQR 1.9–4.3 vs. 5.7 cm, IQR 4.9–7.4 cm, respectively, *p* < 0.01) compared with that of non-AA women.

This study demonstrates similar safety and efficacy of MRgFUS treatment for symptomatic uterine fibroids for all women, regardless of self-reported race. AA women present earlier for treatment with greater symptoms by age and have a larger number and smaller size fibroids. It has already been demonstrated that AA patients have larger uteri and a greater fibroid burden [5]. Our subanalysis at a single institution indicates AA to have a higher screen failure rate due to multiple small fibroids compared with fewer and larger fibroids among non-AA, suggesting a different pattern of disease for AA. Despite the differences in presentation, once they passed screening and were treated with MRgFUS, the two groups do not differ in terms of their need for surgical intervention for failed MRgFUS treatment and in terms of their overall symptom improvement and fibroid shrinkage.

MRgFUS is an ambulatory, non-invasive technique for the treatment of uterine fibroids with a high safety profile [14] that enables patients to return to their routine daily activities rapidly, either on the day of the treatment or the day after [26]. Previous studies showed that high-intensity focused ultrasound energy generated by MRgFUS to ablate uterine fibroids can clinically improve and shrink the fibroid without significant AEs [26]. Other trials have reported significant long-term reductions in fibroid-associated symptoms [14,27].

Considering the prevalence of fibroids in the AA population, the severity of their disease, and the high rates of hysterectomy and associated complications [28-33], it is important to understand that MRgFUS is a viable treatment option for this population from both a medical and a cost perspective. Three studies to date investigated the effect of uterine-preserving treatment for uterine fibroids [33-35], although none evaluated the potential differential effects between racial groups. We observed that at the time of enrollment, AA patients were younger, had more symptomatic fibroids (higher SSS QOL), and presented with different fibroid MR characteristics compared with non-AA patients (Table 1), which is consistent with previous studies [5,34,35].

The need for subsequent alternative treatments was similar for AA and non-AA, and the frequency was similar to those reported elsewhere during the 6 and 12 months post-treatment [36]. Okada et al. reported a 4% re-intervention rate after 6 months and 9% re-intervention rates by 12 months in a population of 228 women treated with MRgFUS in four medical centers in Japan between 2003 and 2006 [36]. Combining the results from the phase III and phase IV studies, both the short-term alternative treatment frequency (<12 months) and long-term alternative treatment rates were relatively high. In both groups, the intervention rates 1 year or less following the treatment was 11.6% compared with the 6.6% reported by Okada et al. [36]. We reported alternative treatment rates of 33.3% and 46.1%, respectively, 2 and 3 years after the clinical studies. Intervention rates did not differ between the groups. The mean NPV achieved for both groups in our study was around 20%, which is similar to the NPV achieved in other early clinical trials [15,36]. This is a very low NPV, compared with the higher rates now being achieved with more current technology [36,37]. Previous studies have noted that higher NPV at the end of the treatment is associated with treatment success. It is important to note that our studies were carried out under restricted clinical trial guidelines leading to these low NPVs and these treatment limitations are not in place today.

The higher screen failure rate among AA found in our study should be the subject of future studies with higher numbers of patients. As our numbers were relatively small, it is possible that our findings were secondary to sample size. Today, as the enrollment criteria are less restrictive, it remains to be seen if the high screen failure rate persists.

## Conclusion

We conclude that MRgFUS treatment is a safe and effective uterine-sparing treatment option for AA women. Further studies including longer follow-up of patients achieving higher NPV at the end of the treatment are needed to assess the long-term outcomes of the procedure among all women classified by race.

## Competing interests

Ronit Machtinger was supported by an award from the Focused Ultrasound Surgery Foundation for a part-time fellowship at the Brigham and Women’s Hospital. Fiona Fennessy received clinical trial support from Insightec. Elizabeth A. Stewart serves as a clinical trial investigator at InSightec and NIH (HD060503); a consultant at Abbott, Bayer, and Gynesonics; and a member of the scientific advisory board at Bayer Healthcare Foundation and received royalties from UpToDate, Johns Hopkins University Press, Massachusetts Medical Society. Clare M.C. Tempany received clinical trial support from Insightec.

## Authors’ contributions

RM drafted the manuscript. FMF participated in the study design, performed the MRgFUS treatments, as well as edited the manuscript. EAS participated in the study design; screened, enrolled, and followed the subjects in both trials; and edited the manuscript. SM and KC designed and conducted the statistical analyses and edited the manuscript. CMT participated in the study design and edited the manuscript. All authors read and approved the final manuscript.
